# Socioeconomic Status and Psychopathic Traits in a Community Sample of Youth

**DOI:** 10.1007/s10802-018-0411-0

**Published:** 2018-03-06

**Authors:** Wendy Zwaanswijk, Mitch van Geel, Paul Vedder

**Affiliations:** Education and Child Studies, Faculty of Social and Behavioral Sciences, Leiden University, Wassenaarseweg 52, 2333 AK Leiden, the Netherlands

**Keywords:** Psychopathic traits, Conduct problems, Socioeconomic status, Family SES, Neighborhood SES

## Abstract

The current study aims to address socioeconomic status (SES) as a moderating variable between psychopathic traits and conduct problems in a sample of 2432 Dutch adolescents (*M*_age_ = 14.50 years, *SD* = 1.67, 56% male). Both family and neighborhood SES were measured, with income as a proxy for the level of SES. There were small but significant positive correlations between the behavioral and interpersonal dimensions of psychopathy and family SES, a small but significant negative correlation between the affective dimension and neighborhood SES, and a small and significant positive correlation between neighborhood SES and the behavioral dimension of psychopathy. Results further showed that the relations between youth psychopathic traits were moderated by neither family SES nor neighborhood SES. The results suggest that the relations between psychopathic traits and conduct problems are equally strong for lower and higher SES youth. Taken together, these findings warrant the conclusions that SES does not play a role as a moderator in the relation between psychopathy and conduct problems.

Psychopathy is a personality disorder that consists of interpersonal, affective, and behavioral characteristics (Hare 2003; Salekin 2016). The interpersonal dimension refers to superficial charm, manipulation, grandiosity, and lying; the affective dimension includes traits such as lack of remorse or shame, shallow emotions, and callousness; and the behavioral or lifestyle dimension is about behaviors such as impulsivity, need for excitement, and irresponsibility (Cooke and Michie 2001). For predictive and treatment purposes it is important to examine the construct of psychopathy in youth, and to examine relations between sociocultural factors and juvenile psychopathic traits (Rubio et al. 2014). This paper focuses specifically on the relation between youth psychopathic traits and family and neighborhood socioeconomic status (SES).

SES is sometimes taken into account as an additional or control variable when examining psychopathic traits and its correlates, but rarely the main object of the study (Mills-Koonce et al. 2016). Though some studies have found negative (e.g., Kahn et al. 2013), or positive relations (e.g., Garcia et al. 2012) between SES and psychopathy, several studies also report non-significant relations between SES and youth psychopathic traits (e.g., Lynam et al. 2007, 2008) which seems consistent with studies that suggest that psychopathic traits are substantially genetic in nature, and that shared environmental influences in the development of psychopathic traits are small (e.g., Bezdjian et al. 2011; Blonigen et al. 2005; Larsson et al. 2006; Rhee and Waldman 2002; Viding et al. 2005).

The studies mentioned so far consider direct links between SES and psychopathy, but there is also an increasing number of studies that examine SES as a moderator between psychopathic traits and conduct problems or antisocial behavior. With regard to the moderating role of SES between psychopathic traits and conduct problems there are competing theories and results. The ‘social push’ hypothesis suggests that in lower SES households it would be difficult to find relations between psychopathy and antisocial behaviors because the environmental risk factors that ‘push’ children towards antisocial behavior would likely camouflage relations between psychopathy and antisocial behavior. However, in higher SES environment, with fewer environmental risks, biological predispositions towards antisocial behavior would more likely be detected (Raine 2002). For youth psychopathic traits, the social push hypothesis was supported by a study wherein it was found that psychopathic tendencies were more strongly related to risk decision making among high SES children (Gao et al. 2009), whereas another study found that only the relation between impulsivity and substance abuse was stronger in high SES neighborhoods (Ray et al. 2016).

Another line of reasoning states that the relation between youth psychopathic traits and antisocial behavior would be stronger in lower SES neighborhoods. In higher SES neighborhoods there would be more informal social control and residents willing to intervene in youth antisocial behavior, thus preventing manifestations of antisocial behavior of youth with psychopathic tendencies (Meier et al. 2008; Sampson et al. 1997). This line of reasoning was supported by two studies that found that impulsivity was more strongly related with antisocial behavior in poorer neighborhoods (Lynam et al. 2000; Meier et al. 2008), or that callous-unemotional (CU) traits are more strongly related to delinquency in lower SES neighborhoods (Markowitz et al. 2015; Meier et al. 2008). There are also studies that found no interaction between CU-traits and neighborhood SES on the development of antisocial behaviors or delinquency (Kroneman et al. 2011; Ray et al. 2016; Trentacosta et al. 2009), but, instead found that irrespective of neighborhood SES, psychopathic traits were positively related to antisocial behaviors. This review suggests that if SES does impact on the relation between psychopathic traits and antisocial behavior, neighborhood SES is a more likely candidate than family SES.

## Current Study

The goal of the current study is to study the relation between psychopathy and conduct problems using both neighborhood and family SES as moderators. We add to the existing literature in several ways. The existing literature has been contradictory as to whether the relation between psychopathy and antisocial behavior is stronger for higher SES youth (Gao et al. 2009), lower SES youth (Meier et al. 2008) or equally strong for both (Trentacosta et al. 2009). Not only are studies contradictory, they are also difficult to compare because they have focused on very different populations: eight and nine year old girls (Kroneman et al. 2011), community twins (Gao et al. 2009), justice involved youth (Ray et al. 2016), low income families (Trentacosta et al. 2009), and a community sample from Iowa (Meier et al. 2008). Besides markedly different samples, studies have also differed in their definition of SES with for example Gao et al. (2009) focusing on family SES, and Meier et al. (2008) focusing on disadvantaged and affluent neighborhoods. Furthermore, some studies focused only on parts of psychopathy such as impulsivity (Lynam et al. 2000) or CU-traits (Kroneman et al. 2011). Our study will consider whether SES moderates the relation between psychopathy and antisocial behavior in a large community sample of Dutch youth. We will include all three dimensions of psychopathy (interpersonal, affective, and behavioral) and use both family SES and neighborhood SES. To the best of our knowledge, this will be among the few studies to consider the moderation of SES between youth psychopathic traits and antisocial behavior in a large community sample (also Lynam et al. 2000; Meier et al. 2008) and the only study to analyze these relations in a community sample using all three aspects of psychopathy and two different proxies for SES.

## Method

### Participants

The total sample consisted of 2855 adolescents. Neighborhood income was based on census data retrieved by postal codes of the participants. Therefore, the participants who did not indicate their postal codes (*n* = 155) and participants who gave a non-existing postal code (*n* = 57) were not included in the analyses. In addition, there were participants who only gave the numerals, and no letters of the postal code (*n* = 211), which made it impossible to establish the neighborhood of the participant. Therefore, these participants were also excluded. This resulted in a final sample of 2432 participants residing in 817 different neighborhoods with 1351 boys (56%) and 1081 girls from 21 junior vocational and five senior vocational high schools across the Netherlands. The average age of the sample was 14.50 years (*SD* = 1.67), and there were 41 participants who did not report their age. About 55% was from native-Dutch origin, the other 45% had diverse ethnic backgrounds (e.g., Moroccan-Dutch, Turkish-Dutch, Surinamese-Dutch). Following Statistics Netherlands (2000), we distinguished three groups: 1327 native-Dutch, 168 Western immigrants (e.g., Polish or French), and 937 non-Western immigrants (e.g., Surinamese or Moroccan). A chi-square test revealed that among those who did not provide valid postal codes there were relatively more boys χ^2^ (2, *N* = 2855) = 11.975, *p* = 0.001 (64.5%) than among those that did (55.5%). A t-test revealed that those who did not provide a postal code (*M* = 14.30, *SD* = 1.82) were slightly younger than those who did (*M* = 14.50, *SD* = 1.67), (*t*(2802) = 2.212, *p* = 0.027, Cohen’s *D* = 0.08). Furthermore, those who did not provide a postal code scored slightly higher on conduct problems (*M* = 7.37, *SD* = 1.85) than those who did (*M* = 7.16, *SD* = 1.71), (*t*(554.789) = 2.212, *p* = 0.027, Cohen’s *D* = 0.12). There was no difference in the family SES scores (*t*(2853) = −1.407, *p* = 0.159). Using a MANOVA, we found no differences between the interpersonal, affective, and behavioral dimensions for those who did and those who did not provide a postal code, Wilks’ Lambda *F*(3, 2793), 2.388, *p* = 0.067. Taken together, there were no significant differences between responders and non-responders on psychopathic traits and SES, and only small differences with regard to age and conduct problems, which likely are significant due to a large sample size. However, among the non-responders there were considerably more boys.

### Measures

#### Psychopathic Traits

The Youth Psychopathic traits Inventory (YPI; Andershed et al. 2002) is a 50-item self-report measure to assess the ‘core’ traits of psychopathy in youths from the general population. The measure consists of ten subscales (e.g., Dishonest Charm, Grandiosity, Lying, Manipulation, Remorselessness, Unemotionality, Callousness, Thrill Seeking, Impulsiveness and Irresponsibility). Participants were asked to indicate to which degree the 50 statements applied to them on a four-point Likert scale, ranging from 1 (*does not apply at all)* to 4 (*applies very well*). Sample items are, “*When I need to, I use my smile and my charm to use others*” for the interpersonal dimension, “*When other people have problems, it is often their own fault, therefore, one should not help them*” for the affective dimension, and “*I get bored quickly by doing the same thing over and over*” for the behavioral dimension. We used the Dutch translation of the YPI (Das and de Ruiter 2003). In a sample of Dutch adolescents in secondary school, YPI total and dimension scores have been positively related to a dominant and hostile interpersonal style (i.e., *r* ranging from 0.11 to 0.30), drug and alcohol use (i.e., *r* ranging from 0.10 to 0.28) for boys and girls separately, indicating adequate construct validity (Hillege et al. 2010). Internal consistencies as estimated with MacDonald’s omega (ω) for the YPI total score and dimension scores were moderate to good. For the total score ω was 0.87, for the interpersonal dimension ω was 0.82, for the affective dimension ω was 0.65, and for the behavioral dimension ω was 0.66.

#### Conduct Problems

The Strengths and Difficulties Questionnaire (SDQ; Goodman 1997) is a short behavioral screening instrument that measures psychosocial adjustment in adolescence. For the present study, the Dutch translation (Van Widenfelt et al. 2003) of the five item conduct problems scale was used with items referring to antisocial behaviors (e.g., *“I take things that are not mine from home, school or elsewhere”*). Participants rated an item on a three-point ordinal scale: (1) *not true*, (2) *somewhat true*, or (3) *certainly true*. In a Dutch sample of adolescents, the SDQ has been shown to have good concurrent validity (Van Widenfelt et al. 2003), with scores of the SDQ show strong correlations with the Child Behavior Checklist and Youth Self-report. Internal consistency of this scale as estimated with MacDonald’s omega was 0.63.

#### Family SES

The Family Affluence Scale (FAS-II; Boyce et al. 2006) was used to examine family SES. It is a brief asset-based measure of family wealth in adolescent surveys, which is internationally used (Currie et al. 2008). This self-report measure consists of four questions, developed so that adolescents could give an approximation of their socioeconomic status. These questions are *Does your family own a car, van, or truck?*; *Do you have a bedroom for yourself?*; *During the past 12 months, how many times did you travel away on holiday with your family?*; and *How many computers does your family own?*. Scores on the FAS have been highly correlated with Gross Domestic Product and national health indicators in adolescent samples in 35 different countries (Boyce et al. 2006). The FAS has been found to be a valid indicator of family SES when asked directly to youth (Currie et al. 2008).

#### Neighborhood SES

Neighborhood membership was determined by the postal codes provided by the participants. In the Netherlands, postal codes consist of four digits and two letters, which were all used to determine the neighborhood of the participant. Through this level of specificity postal code neighborhoods are generally made up of one or a couple of connected streets that have commonly the same type of living, attracting the same type of households. Postal code neighborhoods should not be confused with US zip code regions, which may represent far more heterogeneous living quarters and households. Neighborhood income, which was used as a proxy for neighborhood SES, was provided by Statistics Netherlands (CBS; www.cbsinuwbuurt.nl). Neighborhood income was defined as the average income per income receiving resident in a particular neighborhood area.

### Procedure

Schools across the Netherlands were approached for participation. Parents of participants in junior vocational high schools were asked to complete and sign an informed consent form before their children could participate in the study. Participants from senior vocational high schools were all over 16 years of age, and hence signed their own consent form. Of all adolescents and their parents who were asked for participation, 3% declined to participate. The questionnaire was digitally administered in a classroom setting. Before completing the questionnaire students received a short instruction explaining the research aims. In addition, students were informed that completing the questionnaire was voluntary and anonymous. Two members of the research team were always present during the administration in order to answer questions and solve possible computer problems. The teacher was present but not directly involved in the administration. The Institutional Review Board of Ethics at Leiden University approved of the study.

### Statistical Analyses

To analyze whether the relation between youth psychopathic traits and conduct problems was moderated by neighborhood or family SES we used multiple regression analyses. All terms were mean centered prior to entry in the regression analyses. Because some neighborhoods consisted of only one respondent we decided against using multilevel analyses and instead used robust standard errors for our multiple regression as described in Hayes and Cai (2007). Both neighborhood and family SES were entered in the regression analyses simultaneously. Because the three dimensions of psychopathy (interpersonal, affective and behavioral) together comprise the total construct of psychopathy, analyses with the total construct of psychopathy and the separate psychopathic dimensions were run separately to prevent issues with dependency and multicollinearity.

## Results

Mean scores, standard deviations and Pearson correlation coefficients are included in Table 1. There were small but significant positive correlations between the Behavioral and Interpersonal dimensions of psychopathy and Family SES, and a small but significant negative correlation between the Affective dimension and Neighborhood SES, and a small and significant positive correlation between Neighborhood SES and the Behavioral dimension of psychopathy. We used multiple regressions to test for the associations between psychopathic traits and the potential interaction between psychopathic traits and Neighborhood and Family SES. For all analyses the tolerance scores were higher than 0.4 and the VIF scores were lower than 2.5, which suggests that there were no problems with multicollinearity.

**Table 1:** Means, standard deviations and intercorrelations for the variables included in this study

	*M* (*SD*)	1.	2.	3.	4.	5.	6.
1. Family SES	10.493 (1.687)						
2. Neighborhood SES^a^	21.868 (5.126)	0.304***					
3. Interpersonal	1.713 (0.514)	0.071***	−0.018				
4. Affective	1.989 (0.457)	−0.015	−0.098***	0.587***			
5. Behavioural	2.109 (0.501)	0.051*	0.045*	0.626***	0.453***		
6. Psychopathy total	1.937 (0.414)	0.045*	−0.025	0.885***	0.796***	0.834***	
7. Conduct problems	7.160 (1.714)	−0.022	−0.088***	0.506***	0.435***	0.527***	0.584***

*p < 0.05; **p < 0.01; ***p < 0.001

^a^Neighborhood income × 1.000 euros

In the first step for the analysis on psychopathic traits and SES we entered Neighborhood SES, Family SES and the three psychopathy dimensions in a multiple regression analysis; together, these traits explained a significant amount of variance in conduct problems, *R*^2^ = 0.35, *F*(5, 2413) = 202.476, *p* < 0.001. In the second step we included Family and Neighborhood SES, three psychopathy dimensions and the interaction effects between Family SES and Neighborhood SES and the psychopathy dimensions. These variables together explained a significant amount of variance in conduct problems, *R*^2^ = 0.36, *F*(11, 2407) = 97.541, *p* < 0.001. The Interpersonal (*b* = 0.69, *p* < 0.001), Affective (*b* = 0.58, *p* < 0.001) and Behavioral (*b* = 1.12, *p* < 0.001) dimensions were all significant positive predictors of Conduct problems. However, interaction terms between the psychopathy dimensions and either Neighborhood or Family SES were not found significant. The results of the multiple regression analysis using the psychopathy dimensions are summarized in Table 2.

**Table 2:** Results of the multiple regression analyses testing the relations between family and neighborhood SES, three psychopathy dimensions and conduct problems

	*B*	*SE*
Step 1		
Family SES	−0.03	0.02
Neighborhood SES	−0.03***	0.01
Interpersonal	0.70***	0.09
Affective	0.58***	0.08
Behavioral	1.12***	0.08
Step 2		
Family SES	−0.03	0.02
Neighborhood SES	−0.02***	0.01
Interpersonal	0.69***	0.09
Affective	0.58***	0.08
Behavioral	1.12***	0.08
Interpersonal * FSES	0.08	0.06
Affective *FSES	−0.01	0.05
Behavioral *FSES	−0.07	0.05
Interpersonal* NSES	−0.02	0.02
Affective *NSES	−0.01	0.02
Behavioral *NSES	0.01	0.02

*p < 0.05; **p < 0.01; ***p < 0.001

In the first step for the analysis on the total score for Psychopathy and SES we entered Neighborhood SES, Family SES and the total score for Psychopathy in a multiple regression analysis; together, these traits explained a significant amount of variance in Conduct problems, *R*^2^ = 0.35, *F*(5, 2419) = 313.815, *p* < 0.001. In the second step we included Neighborhood SES, Family SES, the total score for Psychopathy and the interaction effects between Family SES and Neighborhood SES and the Psychopathy total score. These variable together explained a significant amount of variance in conduct problems, *R*^2^ = 0.35, *F*(5, 2417) = 220.692, *p* < 0.001. The Psychopathy total score was a significant predictor of conduct problems (*b* = 2.42, *p* < 0.001), however interactions between the total Psychopathy score and Family SES or Neighborhood SES were not significant. Results are reported in Table 3.

**Table 3:** Results of the multiple regression analyses testing the relations between family and neighborhood SES, the psychopathy total score and conduct problems

	*B*	*SE*
Step 1		
Family SES	−0.03	0.02
Neighborhood SES	−0.02***	0.01
Psychopathy	2.42***	0.08
Step 2		
Family SES	−0.03	0.02
Neighborhood SES	−0.02***	0.01
Psychopathy	2.42***	0.08
Psychopathy * FSES	0.01	0.06
Psychopathy * NSES	−0.01	0.02

*p < 0.05; **p < 0.01; ***p < 0.001

## Discussion

The aim of the current study was to examine the relations between both family and neighborhood SES on the one hand and psychopathic traits on the other. Also the study set out to analyze SES as a moderator in the relation between psychopathic traits and conduct problems. Neither family SES nor neighborhood SES was found to moderate the relation between youth psychopathic traits and conduct problems.

We found small but significant relations between SES and the three psychopathy dimensions, and with the exception of a negative correlation between the affective dimension and neighborhood SES, all significant correlations were positive. There was also a significant positive correlation between Family SES and the Psychopathy total score. Note however that all correlation coefficients between SES and psychopathic dimensions were very small (|*r|* < 0.10), and the correlation coefficients between psychopathy as a whole and indicators of SES were also very small (|*r|* < 0.05); results were likely significant because of a large sample size, but the magnitude of the effect sizes mostly seems to support the repeatedly found result that the effect of shared environmental influences in the development of psychopathic traits is small (e.g., Bezdjian et al. 2011; Blonigen et al. 2005; Larsson et al. 2006; Rhee and Waldman 2002; Viding et al. 2005). It is also important to note that we did not distinguish between primary and secondary psychopathy. Whereas primary psychopathy is theorized to be a heritable deficit in emotional sensitivity, secondary psychopathy is theorized to stem from negative life events, including competitive disadvantage such as low SES (Mealey 1995). Thus, the development of secondary psychopathy traits might be influenced by low SES, which would then be reflected in a higher correlation between secondary psychopathy and SES than would be the case with primary psychopathy.

Though previous studies have suggested moderation, with either the higher SES (Gao et al. 2009) or the lower SES youth (Meier et al. 2008) reporting stronger relations between youth psychopathic traits and antisocial behavior, our study is consistent with a number of other studies that suggest that such moderation does not exist (Kroneman et al. 2011; Ray et al. 2016; Trentacosta et al. 2009). It is worth noticing that studies that report statistically significant moderation effects between youth psychopathic traits and SES typically report small effect sizes (Gao et al. 2009; Meier et al. 2008). Though it is true that moderation effects can be difficult to detect in population research (Meier et al. 2008), we would also argue that journals favor statistically significant results and that non-significant results are more likely to remain unpublished (Simonsohn et al. 2014). Ultimately, the tendency to more easily publish significant results may lead researchers and practitioners to overestimate results. In the case of psychopathy, this could lead to an erroneous focus on higher or lower SES youth in intervention and prevention efforts, even though both lower and higher SES youth with psychopathic traits are more likely to report conduct problems. Taking into consideration that reported significant findings have been small and that now several rigorous studies have reported no moderation of SES, in spite of publication bias, the conclusion that SES is not a moderator in the relation between youth psychopathic traits and antisocial behavior seems warranted.

Although overall the evidence suggests that moderation by SES does not play a role in the relation between psychopathic traits and conduct problems, we nevertheless do point out that the study by Meier et al. (2008) found that the relation between youth psychopathic traits and antisocial behavior was stronger for lower SES youth than for higher SES youth. This study was conducted in the United States. Our study, as well as the study by Kroneman et al. (2011), were conducted in the Netherlands and neither found a moderation effect. As stated before, the Netherlands is a welfare state with more accessible healthcare and welfare benefits than the United States (see for example Shorto 2009). Furthermore, violent crime rates are far lower in the Netherlands than in the United States (Nationmaster 2017). The processes of neighborhood disintegration that Meier et al. (2008) present as a possible explanation for their results are likely far less prominent in the Netherlands than in the United States (see also Helliwell et al. 2017).

### Limitations and Future Research

Income was used as a proxy for neighborhood SES, which is a limited measure of neighborhood SES. Other measures, such as unemployment rate and level of education of the residents of a neighborhood, or neighborhood risk factors such as access to drugs, were not taken into account. On the other hand, neighborhood income is a relatively easy marker for policy makers, and defines an easy to target risk group, thus there are also advantages to using only neighborhood income (Markowitz et al. 2015). Using only self-reports for the assessment of psychopathy is a limitation of the current study. Lying and manipulation are characteristics of psychopathy, which makes it questionable whether they will report truthfully when completing the self-report (Lilienfeld and Fowler 2006). A final limitation is that we did not distinguish between primary and secondary psychopathy. To the best of our knowledge, no prior studies on the moderation of SES in the relation between SES and conduct problems has included such a distinction. However, because secondary psychopathy develops due to negative environmental circumstances (Karpman 1948), it is most likely the secondary variant of psychopathy that is related to SES (Mealey 1995). Furthermore, primary and secondary psychopathy have been distinguished using measures of anxiety, and secondary psychopaths have been shown to show higher levels of anxiety compared to primary psychopaths (Skeem et al. 2003). Youth with secondary psychopathy traits might therefore respond differently to (negative, anxiety inducing) environmental influences such as low SES. With a design in which this possible anxiety arousing effect of SES would have been taken into account we might have found moderation effects of SES. Thus, this distinction of primary and secondary psychopathy in relation to SES is important for future research.

### General Conclusion

We found that the relations between youth psychopathic traits and conduct problems were not moderated by family or neighborhood SES. There were statistically significant relations between youth psychopathic traits and conduct problems, though the effect sizes of these relations were small. We used a methodologically rigorous design with a large sample size, well-validated self-report measures, and two separate measures for SES. We also accounted for the multilevel structure of the data by using robust standard errors. Consistent with previous research (Kroneman et al. 2011; Ray et al. 2016; Trentacosta et al. 2009), we found that psychopathy dimensions are predictors of conduct problems, but that SES does not likely affect the development of psychopathic traits, nor does it moderate the relations between psychopathy or conduct problems. Taken together this suggests that if the goal is to screen which youth are at a heightened risk for conduct problems, focusing on psychopathic traits may be worthwhile. Furthermore, the results of the current study suggest that such screening could be useful in both affluent and less affluent youth.
